# Being Attractive Brings Advantages: The Case of Parrot Species in Captivity

**DOI:** 10.1371/journal.pone.0012568

**Published:** 2010-09-07

**Authors:** Daniel Frynta, Silvie Lišková, Sebastian Bültmann, Hynek Burda

**Affiliations:** 1 Department of Zoology, Faculty of Sciences, Charles University in Prague, Prague, Czech Republic; 2 Faculty of Biosciences, Department of General Zoology, University of Duisburg-Essen, Essen, Germany; University of Jyväskylä, Finland

## Abstract

**Background:**

Parrots are one of the most frequently kept and bred bird orders in captivity. This increases poaching and thus the potential importance of captive populations for rescue programmes managed by zoos and related institutions. Both captive breeding and poaching are selective and may be influenced by the attractiveness of particular species to humans. In this paper, we tested the hypothesis that the size of zoo populations is not only determined by conservation needs, but also by the perceived beauty of individual parrot species assessed by human observers.

**Methodology/Principal Findings:**

For the purpose of data collection, we defined four sets of species (40 parrots, 367 parrots, 34 amazons, 17 macaws). Then, we asked 776 human respondents to evaluate parrot pictures of the selected species according to perceived beauty and we analyzed its association with color and morphological characters. Irrespective of the species set, we found a good agreement among the respondents. The preferred species tended to be large, colorful, and long-tailed.

**Conclusions/Significance:**

We repeatedly confirmed significant, positive association between the perceived beauty and the size of worldwide zoo population. Moreover, the range size and body size appeared to be significant predictors of zoo population size. In contrast, the effects of other explanatory variables, including the IUCN (International Union for Conservation of Nature) listing, appeared insignificant. Our results may suggest that zoos preferentially keep beautiful parrots and pay less attention to conservation needs.

## Introduction

Parrots are attractive, colorful birds [1], capable of vocal learning [2] and extraordinary cognitive skills [3]–[6], including numerical competence [7], tool use [8], and imitation [9], [10]. Consequently, parrots belong to the most frequently kept and bred bird order in captivity (cf. [11]). In contrast, natural populations of many parrot species are considerably endangered – 27% species of parrots are listed as threatened and an additional 11% as nearly threatened [12]; cf. [13]. Captive keeping and breeding increases the risk of poaching for the illegal pet market [14]–[18]. In contrast, if properly managed by conservational institutions and respectable private breeders, supporting backup populations are potentially important in the time of unexpected crisis in nature. Parrots raised in captivity can be successfully reintroduced [19]–[21], but see [22]. The potential usefulness of parrots kept by breeders for possible rescue programs is, nevertheless, limited by extremely skewed representation of individual species in both institutional and private collections. Moreover, most private breeders are not interested in keeping endangered, but unattractive, species without commercial value that provide no prospect for sustainable funding of the breed [23]. Because of this, rescue programs involving captive breeding managed mostly by zoos and related institutions contribute substantially to the survival of some species (e.g., *Amazona versicolor*; [24]). Successful reintroduction of Puerto Rican parrots (*Amazona vittata*) may serve as an example [25]–[27]. Parrots kept by zoos and other public institutions are of fundamental importance and the size of worldwide zoo populations may be treated as a simplified measure of ex situ conservation effort. However, long-term captive management of endangered animals is limited by space available for breeding programs in zoos, and single species compete for their share [28]. To be effective, the selection of captive species should take into account case-specific factors such as the availability of habitat for reintroduction of the particular species, their status on the IUCN (International Union for Conservation of Nature) red list, and their capability of breeding in captivity. Still, zoos seem to preferentially shelter species that are large and attractive, even if they are expensive to keep, breed relatively poorly, and are hard to return to the wild [29]. Financial reasons could lead zoos to make such choices to attract visitors who prefer charismatic megafauna [30], but the investment to the exhibits of larger animals make no greater returns than for those of smaller animals [31], [32]. Thus, it seems that it is the very human preference for attractive animals that decides the species selection for captive breeding.

The aim of this paper was to test the hypothesis that the size of zoo populations is not only determined by conservation needs, but predominantly by human aesthetic preferences towards particular species. For this purpose we (1) selected different sets of parrot pictures and asked human respondents to evaluate perceived beauty of each species, (2) analyzed the effect of morphological traits, such as coloration, body size and shape, on these estimates of human preferences, and finally (3), attempted to explain worldwide zoo population size by a set of factors including both perceived beauty and conservation status.

## Materials and Methods

### Ethics Statement

The experiments were performed in accord with the European law and were approved by The Institutional Review Board of Charles University, Faculty of Science. All respondents provided us a written informed consent and agreed to participate in the project voluntarily.

**Figure 1:**
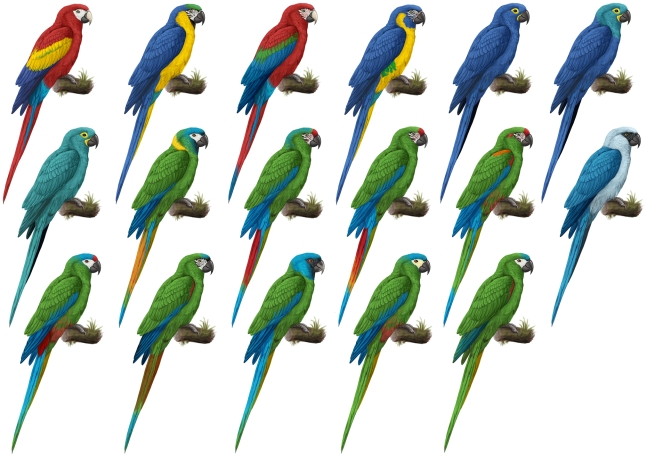
The standardized pictures of 17 macaw species. They are arranged in rows according to perceived attractiveness from the most preferred (top left) to the least preferred (bottom right) species by human respondents.

The aesthetic attractiveness of the species was examined by presenting pictures of individual parrot species to human respondents. For the purpose of data collection, we defined the following four sets of species:

1. Reduced set consisting of only 40 species was adopted to avoid eventual habituation of the respondents and thus maximize precision of the assessment. In order to choose species covering the whole range, from the most represented to those absent in zoo collections, we selected them as follows. First, we divided all parrots into eight groups, according to their numerical representation in zoos: 1,000 and up, from 201 to 1,000, from 101 to 200, from 51 to 100, from 26 to 50, from 11 to 25, from 1 to 10, and 0 individuals. In each group, 5 species were randomly selected using True Random Numbers Generator [33], but inclusion of more than one species belonging to a single genus within the category was avoided. In addition, as only 5 species were kept in numbers exceeding 1,000 individuals, they were all included in the reduced set.
2. Complete set consisting of 367 extant species/subspecies was adopted to maximize taxonomic resolution. It is based on the full list of parrot species [34], supplemented by 11 subspecies characterized by coloration apparently contrasting with that of nominotypic subspecies. Three additional taxa recognized by zoos were included (*Barnardius barnardi, Platycercus flaveolus, Trichoglossus rubritorquis*) and another two taxa were merged with its sister forms (*Cyanoramphus forbesi, Cyanoramphus malherbi*).
3. A set of amazons was introduced to examine morphologically and ecologically homogenous group of parrots. It consists of 34 taxa belonging to the genera *Amazona* (33 taxa) and *Alipiopsitta* (*A.xanthops*), covering all extant species of amazons including those subspecies characterized by a distinct coloration.
4. Macaws: 17 extant species of five genera (*Ara*, *Orthopsittaca, Primolius*, *Anodorhynchus*, *Cyanopsitta, Diopsittaca*) were included because of similar reasons as the amazons; moreover, this small group exhibits considerable color variation (see Fig. 1), and encompasses species highly represented in zoos as well as those that are kept rarely.

The parrot pictures of the reduced set were adopted alternatively from Forshaw and Knight ([35]; further referred as variant 1), Juniper & Parr ([36]; variant 2) and del Hoyo et al. ([1]; variant 3); the second source was also used for the complete set. In order to avoid possible effects of body position, size, and background on rating, the pictures were adjusted with white background, turned right, and resized so that the pictured parrots were of a similar relative size. In the case of amazons and macaws, the pictures were repainted (by S. L.) to fit the precisely identical silhouettes to remove the effects associated with body position, “facial expression”, and shape (Fig. 1). Juniper & Parr [36] served as a reference for the paintings.

Because the number of included species differed considerably among the examined sets, we employed two alternative strategies for the assessment of human preferences. The first one, which we further refer to as Ranking [37], [38], maximizes the informative content by covering the full ordination scale. It requires simultaneous presentation of all pictures to the respondent to allow relative comparisons, so it is hardly applicable to large sets. In contrast, the second assessment strategy, further referred to as Scoring, provides only limited scoring scale. But it benefits from the possibility to present pictures to the respondent consecutively. Such a presentation enables evaluation of extensive sets of pictures.

The reduced set was assessed by both procedures mentioned above, to verify their mutual correspondence. The respondents of the Ranking procedure were Czech citizens, mostly 19–29 years old. Each person was exposed to one set, i.e. 40 pictures, placed on a table in a random assemblage. Then we asked them: “Please, stack the photographs in an order corresponding to the beauty of the depicted parrot, from the most beautiful to the least beautiful one.” The order of the photograph in the pack was then coded by numerals from 1 (the most beautiful one) to 40, further referred to as ranks. Although no explicit time limit was given, all the respondents performed the task within a few minutes. Altogether, we gathered data from 210 respondents; each of the three picture set variants was evaluated by 30 males and 40 females.

Alternatively, Open-Source Software LimeSurvey [39], running on a web server, was used to collect data from 316 respondents (133 men and 183 woman), mainly the students and employees of the Duisburg-Essen University (in Germany). Each respondent was shown the set of 40 parrot pictures (variant 1) in a set order, assigning each of them numbers from 0 (the least attractive) to 6 (the most attractive). Later on, we inverted this seven point scale to obtain values conforming polarity of the other data sets. Furthermore, the respondents were asked to indicate whether they know the pictured parrot or not. The total number of “yes” answers in each species was evaluated as the percentage of knowledge of the parrot. To analyze the effect of the order in which the illustrations were shown, we included one species (*Agapornis fischeri*) twice – in the fourth and forty-first sequence of the screening.

The complete set of species was evaluated by 112 respondents in the Czech Republic (56 men and 56 women). Each respondent was asked to evaluate each of 367 parrot species presented on a computer screen in a random order. At the beginning of the session, the first block of 35 species appeared on the screen as thumbnails arranged six by six on consecutive screens, to provide the respondent with basic information about variance in appearance of evaluated parrots. Then, the respondent was asked to score larger pictures (360×540 pixels), appearing one after another on the screen, on a five point scale (1 corresponding to the best). The timing of presentation was determined by the respondents themselves as the picture on the screen was replaced by another one when they successfully entered the score. The process was repeated until the last species was scored. Next, we standardized raw scores by subtracting respondent's mean score and dividing by its standard deviation. Because species means of raw scores were highly correlated with standardized ones (r^2^ = 99.5%, p<0.0001), we further analyzed the raw variables as they were more intuitive.

The sets of amazons and macaws were evaluated by 65 (30 men and 35 women) and 73 (32 men and 41 women) respondents by ranking method.

All respondents agreed to participate in the project voluntarily. Each subject provided a written informed consent and additional information about gender, age, experience with parrots, and knowledge of the presented species.

Information about the numbers of individuals of each particular parrot species kept in zoos worldwide was obtained from the ISIS [40] online database (http://www.isis.org), accurately covering [41] more than 700 zoos and aquariums from 72 countries.

Listing of species in the IUCN categories “Nearly Threatened”, “Vulnerable”, “Endangered”, and “Critically Endangered” ([34], cf. IUCN website http://www.iucnredlist.org.), was coded as “present on the list”, while the category “Least Concern” was coded as “not present”. The number of species inside each parrot genus was used as a simplified measure of taxonomic uniqueness of the species. Standard body measurements (total, wing, tail, beak, and tarsus length) of each species were taken from Juniper and Parr [36], del Hoyo et al. [1], and/or Arndt [42]. We extracted principal components from these log transformed traits. The first component, accounting for 88.8% of variation, is further referred as body size, while the second one (7.7%), which may be interpreted as relative tail length, as body shape. Supplementary information was obtained from Robiller [43]. The sizes of species ranges (further referred to as range size) were extracted from graphical maps in Juniper and Parr [36]. The presence/absence of the following colors on parrot bodies was recorded: blue, green, red, orange, yellow, purple/pink, black, and white.

### Statistical analyses

In order to quantify and test congruence in species ranking provided by different respondents, we adopted Kendall's Coefficient of Concordance. Prior further analyses, the raw ranks were transformed as follows: each value was divided by the number of evaluated species (40) and square-root arcsin transformed. The variables showing lognormal distribution (number of individuals kept in zoos, body measurements, taxonomic uniqueness, range size) were transformed by natural logarithm prior to the analyses. Principal Component Analysis (PCA) was performed to visualize the multivariate structure of the data sets. ANOVA/MANOVA, Hotelling tests, GLMs and/or Multiple regression analysis were applied to test the effects of independent explanatory variables. Mann-Whitney test was used as a non-parametric alternative for variables deviating from normality (raw sores).In order to partially control the effects of phylogeny, we divided the studied species into 10 clades (Nestor-Strigops; Cacatuidae; Psittrichas; Psittacini; amazons and allies of Arini; macaws and allies of Arini; Psittaculini; Loriinae, main branch of Platycercini; Neophema-Agapornis and allies) and introduced clade as a random factor into GLMs. The clades were defined according to Wright et al. [44]; putative phylogenetic position of the remaining genera was set according to conventional taxonomy [1]. Three species suspected to be actually extinct (*Anodorhynchus glaucus, Charmosyna toxopei, C.diadema*) were excluded from all analyses dealing with size of zoo populations. We performed most calculations in Statistica 6.0. [45] and SPSS v.16.0 [46].

## Results

### Agreement among respondents and methods

#### Reduced set

The results of the ranking procedure revealed considerable congruence among the respondents in all variants of the reduced set consisting of 40 species. Kendall's Coefficients of Concordance W were 0.258, 0.239, 0.231, and 0.197 for the variants 1, 2, 3, and pooled data, respectively (all p<0.001). Mean transformed ranks computed for individual variants were mutually highly correlated (r^2^ = 61.2%, 39.5%, and 55.0% for 1 vs 2, 1 vs 3, and 2 vs 3 respectively; all p<0.0001). The correlations between mean transformed ranks provided by male and female respondents were even higher: r^2^ = 85.2 (70.9, 88.4 and 73.4 for variants 1, 2 and 3, respectively).

Nevertheless, Manova revealed small, but significant effect of both variant (F_78,332_ = 5.76, p<0.0001) and gender (F_39,166_ = 1.81, p = 0.0056). Separate ANOVAs performed in individual parrot species (Bonferoni corrected Ps<0.05) revealed no effect of gender, but confirmed the effect of the variant in 13 species. Post hoc tests revealed that *Nymphicus hollandicus* and *Chalcopsitta cardinalis* were more preferred in variant 1 than in variant 3, while the opposite was true for *Enicognathus leptorhynchus*, *Ara glaucogularis*, *Psephotus dissimilis*, *Geopsittacus occidentalis*, *Touit melanonota*, and *Eunymphicus cornutus.* When variants 2 and 3 were compared, *Agapornis canus*, *A. fischeri*, and *Loriculus philippensis* were more preferred in the former while *Pionus fuscus*, *Touit melanonota*, and *Eunymphicus cornutus* in the latter; finally, *Geopsittacus occidentalis* and *Loriculus philippensis* were more preferred in variant 2 than in variant 1.

Scoring procedure confirmed agreement among the respondents (W = 0.246, n = 316, p<0.001), as well as high positive correlation between mean preferences exhibited by men and women (r^2^ = 91.7%; p<0.0001). Mann-Whitney tests revealed significant (p<0.05, Bonferoni adjusted) effect of gender on preference in two species out of 39 examined ones. Both *Agapornis fischeri* and *Psittaculirostris edwardsii* were more preferred by women than men. Mean scores of individual species closely correlated with corresponding mean ranks obtained by ranking procedure (variant 1): r^2^ = 81.9% (p<0.0001).

#### Complete set

The scores obtained for the complete set of 367 pictures also revealed sufficient congruence among the respondents (PC1 explains 17.3% of total variation). The correlation of species means with mean ranks obtained for the corresponding 40 species set, containing the identical pictures (variant 2), was high: r^2^ = 84.5% (p<0.0001).

#### Amazons

Congruence among the respondents was less pronounced, but still statistically significant (W = 0.157, n = 65, p<0.001). Preferences were affected by gender (Hotelling test: T2 = 197.80, n males = 30, n females = 35, F_33,31_ = 2.95, p<0.0016): men preferred *A. guildingii,* while women *A. viridigenalis* (Bonferoni adjusted t-tests at α = 0.05). Nevertheless, preference ranks of individual species provided by men and women were correlated (r^2^ = 21.8%; p = 0.0053). Mean transformed ranks of amazons species were not correlated with mean scores of corresponding species obtained for the complete set (r^2^ = 6.6%; p = 0.1425).

#### Macaws

Congruence among the respondents was high (standardized; W = 0.287, n = 72, p<0.001) and no effect of gender on human preferences was found by multivariate Hotelling test (T2 = 14.60, n males = 32, n females = 41, F_16,56_ = 0.72, p = 0.7622). Mean transformed ranks of particular species of macaws were correlated with mean scores of corresponding species obtained for the complete set (r^2^ = 56.9%; p = 0.0005).

### Traits associated with human preference

The complete set was large enough to assess the effects of particular colors on human preferences. For this purpose, we performed GLM in which preference scores were taken as dependent variable and presence of red, orange, yellow, green, blue, pink-purple, white and black colors as well as body size and shape as explanatory variables. This model (r^2^ = 29.5%) revealed that what is more preferred are parrots characterized by large body size (β = −0.214; F_1,358_ = 19.3, p<0.0001) and long tail (β = −0.370; F_1,358_ = 65.7, p<0.0001), and those having blue (β = −0.163; F_1,358_ = 12.8, p = 0.0004), orange (β = −0.147; F_1,358_ = 10.5, p = 0.0013), and yellow (β = −0.145; F_1,358_ = 10.3, p = 0.0014) colors. On the contrary, green parrots tended to be less preferred (β = 0.097; F_1,358_ = 4.0, p = 0.0474).

### Correlates of worldwide zoo-population size

#### Reduced set

**Figure 2:**
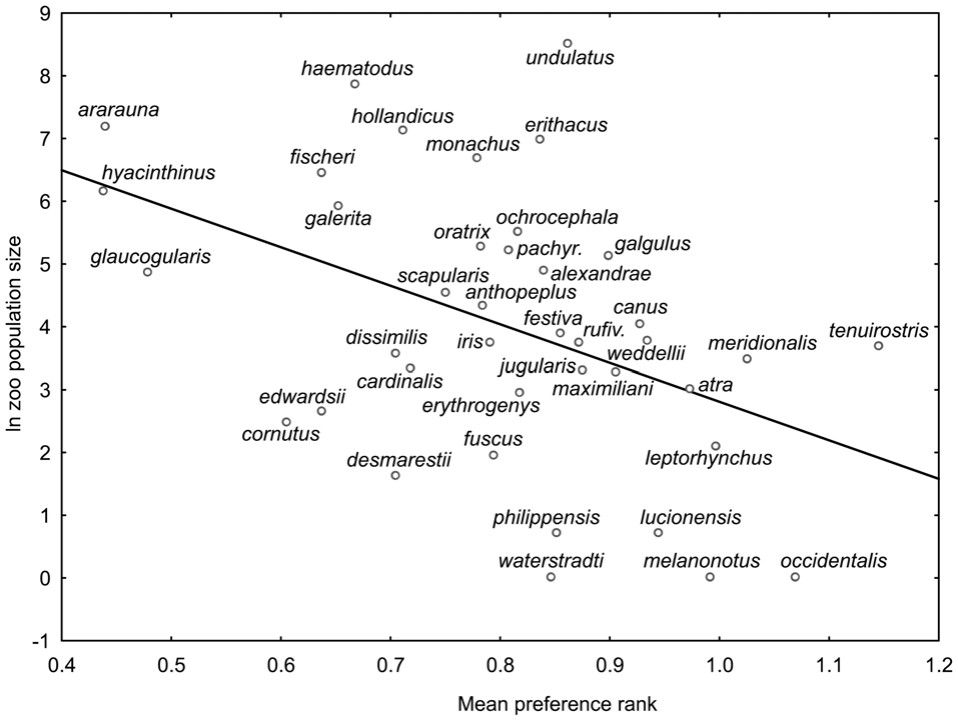
Preference ranks of the reduced parrot picture set. The figure shows the relationship between mean preference rank of parrots (variants of pictures pooled) and its worldwide zoo population size in the reduced set of 40 species (R^2^ = 19.9%). The higher the rank, the lower the human preference of the species is.

**Figure 3:**
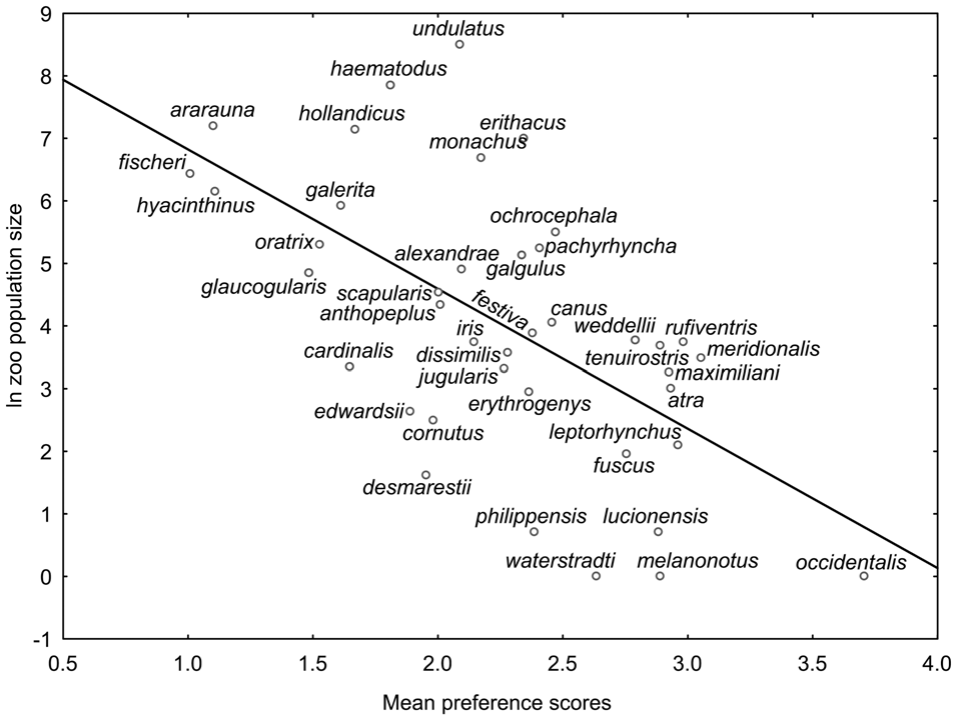
Preference scores of the reduced parrot picture set. The figure shows the relationship between mean preference scores of parrots (picture variant 1) and its worldwide zoo population size in the reduced set of 40 species (R^2^ = 37.2%). The scale of scoring ranged from 0 to 6. The higher the mean score, the lower the human preference of the species is.

We found significant positive correlation between the number of individuals kept in zoos worldwide and human preference ranks (Variant 1: r^2^ = 38.2%, p<0.0001; Variant 2: r^2^ = 14.3%, p = 0.0162; Variant 3: r^2^ = 4.1%, p = 0.2118; pooled variants 1–3: r^2^ = 19.9%, p = 0.0039, see Fig. 2 and Fig. 3), as well as with mean scores (Variant 1: r^2^ = 37.2%, p<0.0001) among 40 parrot species. When we applied partial correlation to remove the effect of foreknowledge (i.e., proportion of respondents who marked the particular species as known), the relationship between mean scores and zoo population size remained significant (r^2^ = 13.7%, p = 0.021).

In order to also examine the effects of other factors on zoo population size, we performed GLMs. The initial full model included preference ranks (computed from pooled variants), range size, body size, body shape, and IUCN listing as explanatory variables, and it revealed significant effects of the former two factors only. Final model explained 43.8% of variation in zoo population size: preference rank (β = 0.422; F_1,37_ = 11.4, p = 0.0017) and range size (β = 0.476; F_1,37_ = 14.5, p = 0.0005).

#### Complete set

**Figure 4:**
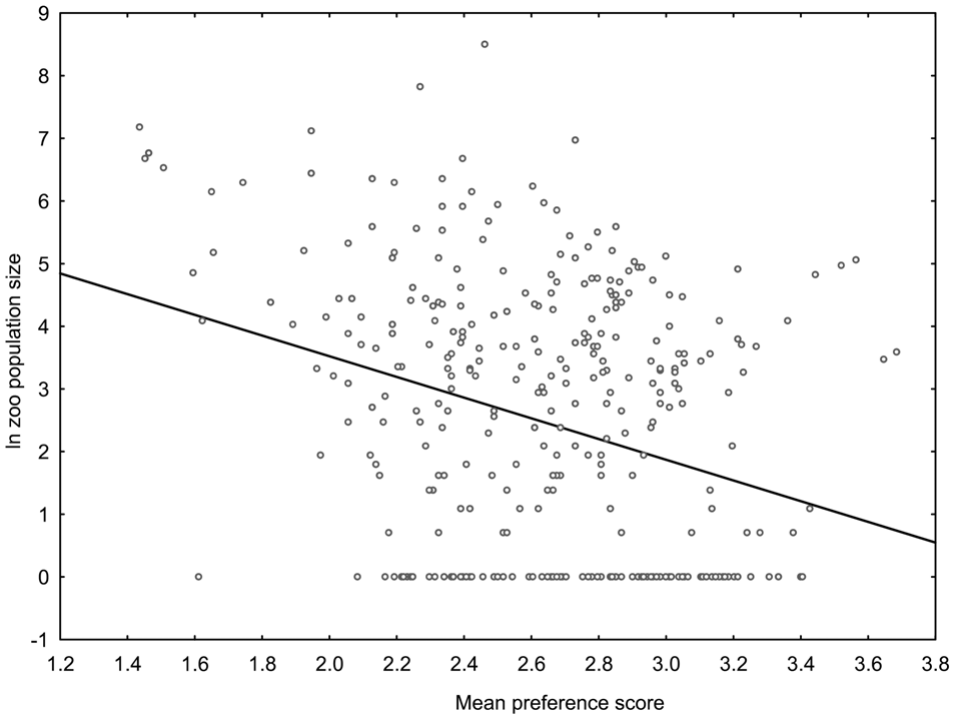
The complete set of 367 parrot pictures. The figure shows the relationship between mean preference scores of parrots (picture variant 2) and its worldwide zoo population size in the complete set of 367 species (R^2^ = 9.2%). The scale of scoring ranged from 1 to 5. The higher the mean score, the lower the human preference of the species is.

When all 367 species were included, the correlation between mean scores of human preference and the number of individuals kept in zoos worldwide decreased to r = 0.304 (r^2^ = 9.2%, p<0.0001, Fig. 4). Nevertheless, 16 of the 18 ( = 5%) most preferred parrot species were kept in numbers exceeding 50 individuals. Zoo populations exceeding this value were recorded in 98 out of 367 extant species only.

Next, additional explanatory variables were included and GLM performed. No effect of taxonomic uniqueness (F_1,348_ = 2.8, p = 0.0978) and IUCN listing (F_1,348_ = 2.1, p = 0.1435) was found, so these variables were excluded. The reduced model (r^2^ = 44.9%) included mean scores of human preferences (β = −0.264; F_1,350_ = 28.8, p<0.0001), range size (β = 0.415; F_1,350_ = 94.2, p<0.0001), body size (β = −0.352; F_1,350_ = 42.7, p<0.0001), and body shape (β = 0.146; F_1,350_ = 6.7, p = 0.0099). The effect of clade, treated as a random factor, was also significant (F_9,350_ = 4.7, p<0.0001).

#### Amazons and macaws

**Figure 5:**
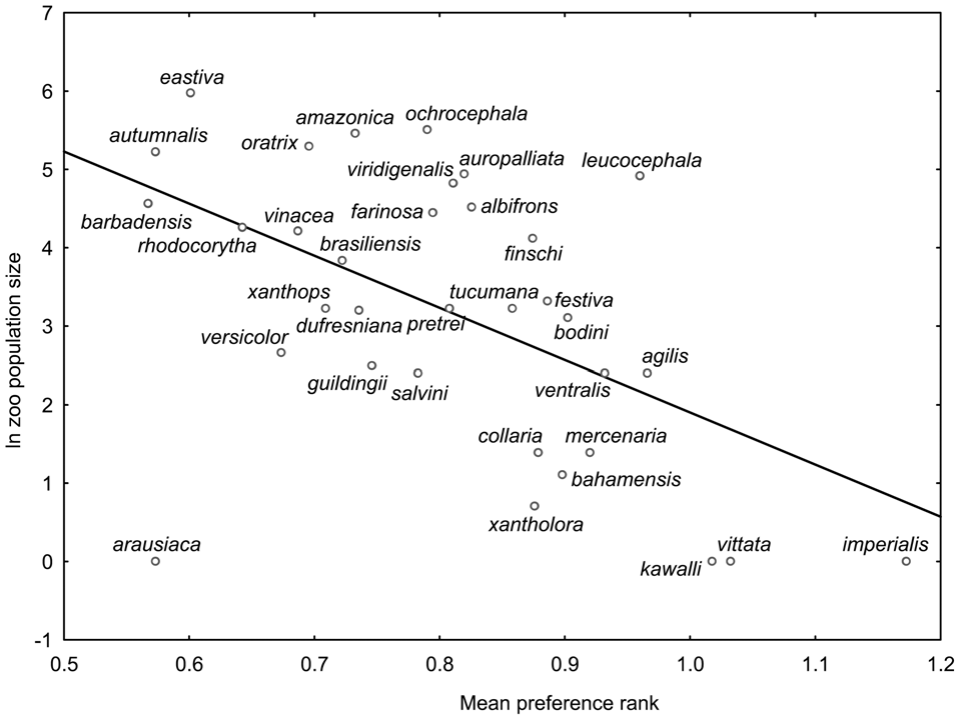
The amazons. This figure shows the relationship between mean preference rank of amazons (shape adjusted pictures) and its worldwide zoo population size (34 species/subspecies; R^2^ = 28.1%). The higher the rank, the lower the human preference of the species is.

**Figure 6:**
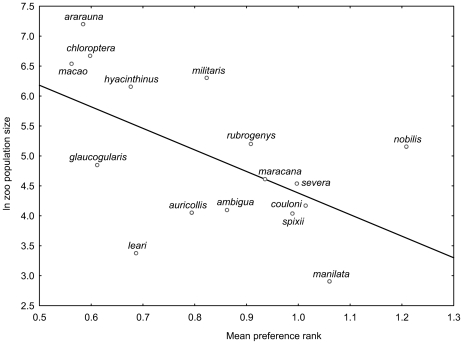
The macaws. This figure shows the relationship between mean preference rank of macaws (shape adjusted pictures) and its worldwide zoo population size (16 species/subspecies; R^2^ = 31.6%). The higher the rank, the lower the human preference of the species is. Mean preference rank of the extinct *Anodorhynchus glaucus* is 0.81.

In amazons, the number of individuals kept in zoos worldwide was correlated with preference ranks of individual species (n = 34; men: r^2^ = 13.6%, p = 0.0321; women: r^2^ = 21.1%, p = 0.0063; genders pooled: r^2^ = 28.1%, p = 0.0013; Fig. 5). In macaws, this correlation was positive as well (n = 16; r^2^ = 31.6%, p = 0.0235; Fig. 6).

## Discussion

We found a fairly good agreement among the respondents in aesthetic preferences towards pictures of parrot species. In this respect, there were no substantial differences between the sets of pictures representing the whole diversity of parrots (complete and reduced set) and those covering just a small clade, such as macaws or amazons. Nevertheless, the respondents' agreement was the lowest in the case of amazons who are highly homogenous in their morphology, coloration, and pattern, and the respondents repeatedly expressed complaints about similarity of evaluated pictures within this set.

We were not surprised much by the agreement among the respondents evaluating relatively small sets of pictures by ranking method. In our previous papers, we used the same method for evaluation of human preferences within various vertebrate taxa [37], [47], including some birds [48], and we found comparable results. In contrast, we expected that the respondents might be confused by extremely extensive sets, but the respondents fairly agreed, even in evaluation of the complete set, consisting of as many as 367 parrot species. Moreover, the resulting mean scores fit well with the ranks obtained by ranking procedure within a reduced set of 40 pictures. This is even more surprising as two methods of evaluation are compared: ranking of real simultaneously presented pictures and scoring of virtual pictures successively shown on screen. But the direct comparison between these evaluation methods, which we carried out in the variant 1 of the reduced set, confirmed that these methods produce nearly equivalent results.

Gender differences in evaluation of parrot beauty were small enough to be omitted in the study analysing the relationship between animal beauty and representation of particular species in zoos worldwide. Zoo curators and visitors belong to both genders, and, thus, decision making is not done exclusively by either one. In this context, pooling the data seems to be adequate, in spite of significant comparisons between the genders. Gender differences in species ranking are, of course, worthy of further examination.

High congruence in evaluation of pictures does not necessarily mean that these pictures reliably represent particular parrot species. We compared human preferences towards 40 parrot species of the reduced set, as assessed using three variants of pictures. Although there was a basic agreement in ranking the species, it was apparently lower than those in the above discussed comparisons, concerning the identical pictures. Thus, reliability of pictures may represent a possible methodological pitfall that potentially decreases precision of human preference estimates. We tried to avoid this problem either by combining the results obtained for different variants of pictures (reduced set) or by repainting the colors and patterns into the same shape (silhouette) of the parrot. The latter approach is, however, applicable exclusively in the case of morphologically homogenous groups as macaws and amazons.

The superstars of our beauty competition tended to be large, colorful and long-tailed parrots, while small and dull (green) parrots received no attention. Visual inspection of the most prominent losers (e.g., *Psittrichas fulgidus, Nestor notabilis, N. meridionalis, Cacatua tenuirostris, Enicognathus leptorhynchus*) suggests that they usually possess an exaggerated, hawk-like beak (curved and sharp), which might be perceived by humans as weaponry. The effect of body size on human preferences may be surprising, considering that the respondents evaluated size-standardized pictures, providing no direct information about the absolute body size of the parrots. Thus, either are large parrot species statistically more beautiful per se, or are the human respondents able to estimate the real body size of the depicted parrots. Allometric component of body shape (already contributing to the first principal component, treated here as a multivariate body size) could play a role in both of these scenarios. Nevertheless, we can not exclude the effect of the respondents' previous knowledge of some depicted species, enabling to predict the body size of similar parrots.

Relationship between human preferences and the size of worldwide zoo population was positive and significant within all four examined sets of parrot species. We previously reported similar relationships within some other taxa of vertebrates as boid snakes [37], basal mammals (monotrems, marsupials, Afrotheria and Xenarthra), Laurasiatheria (comprising mainly of ungulates, carnivors and insectivors), terrestrial birds, and pheasants [48]. This suggests that selective keeping of beautiful species in zoos is a more widespread phenomenon, not exclusive to the parrots.

Correlation between beauty of the species and its representation in zoos does not provide any information concerning the direction of the putative causal relationship responsible for the observed statistical association. Thus, we cannot exclude the alternative hypothesis that the species highly represented in zoos worldwide have better chance to be preferred by the respondents because of their higher rate of prior experience with commonly exhibited species. We argue, however, that typical respondents never met the vast majority of vertebrate species including parrots. When complete species lists of any taxonomic level are evaluated, previous knowledge is too rare to be responsible for the observed correlations. This problem is worthy of further experimental examination.

One can argue that our respondents belong to just a single culture and that perception of beauty may fundamentally differ in people of different cultures and experiences. Nevertheless, our previous study revealed a surprisingly close correspondence between rankings of snake species by people from such different cultures as are those that are in Europe and Papua New Guinea [47]. Our unpublished data also suggest high cross-cultural correspondence in ranking of other vertebrate taxa including parrots (e.g. correlation coefficient between Europe and east of Lesser Sunda Archipelago was r^2^ = 0.38; Frynta, unpublished results).

Proportion of variation in zoo population size attributable to human preferences varied among the studied sets; the highest values were found within macaws (r^2^ = 31.6%) and amazons (r^2^ = 28.1%), while the most relaxed ones were within reduced (r^2^ = 19.9%) and especially the complete (r^2^ = 9.2%) sets. Relatively low percentage, revealed by the analysis of the complete set, may be explained either by lower precision of human preference estimates (only one non-standardized variant of pictures; possible confusion due to large set of evaluated species), or by masking effect of the vast majority of parrot species which are both not especially attractive to humans and poorly but erratically represented in zoo collections. The former explanation suggests that we probably underestimated rather than overestimated the size of the effect, while the latter one emphasizes that a subset of species (e.g., the most beautiful or most represented in zoos) is affected much more than the remaining ones.

Inclusion of additional variables into the model, partially controlled for the effect of phylogeny, revealed that, besides human preferences, body size and range size also contribute to the worldwide zoo population sizes of individual parrot species. The substantial positive effect of animal body size on its representation in zoo collections is an almost universal rule [30]. Such relationships were previously reported in various animal taxa [37], [48]. Body size is an apparent trait for zoo visitors and curators making decisions about which species would be kept and bred. In practice, unlike in our experiments, it is an integral component of parrot attractiveness that cannot be easily separated. Because we adjusted parrot pictures to the same size, our respondents had no direct information on body size of the evaluated species (as discussed above, allometric relationship between body segments may provide some indirect information) and we succeeded in keeping the effect of body size apart.

The larger the geographic range of distribution, the higher the zoo population size of the parrot species is. Widespread parrot species are easier to obtain and import, yet the slope of allometric relationship between zoo population size and distribution range is much smaller than one (0.344; 95%CI = 0.264–0.424). That means species with small distribution range are still relatively overrepresented. This may be interpreted as evidence that zoos tend to keep and breed rare species in their collections preferentially.

In contrast to the above factors, neither IUCN listing nor taxonomic uniqueness, i.e., the variables best reflecting conservation value of the species, had effect on zoo population size. This finding is alarming because zoos seem to pay no systematic attention to species with urgent conservation needs. This conclusion is of course based on the analysis of aggregate data and thus does not imply absence of beneficial rescue programmes managed by zoos. Alternatively, these data may be interpreted, e.g., as an evidence of undesired effect of legal barriers preventing zoos from obtaining species worthy of conservation efforts.

The absence of selective keeping of endangered species by zoos may be attributed to a dual function of zoos and does not necessarily mean the absence of conservation efforts and consequences. The primary function of these institutions is educational and cultural. Successful exposition of not only rare, but also common species improves public views towards animals and may as the so-called flagship species indirectly support conservation efforts of other (similar and/or related) species in need. In spite of this, endangered species may play the same role for visitors as the common ones, while filling the conservation role at the same time. This is in agreement with the ‘Ark’ concept [49] supported by the WAZA (World Association of Zoos and Aquariums) strategy [50]. Because zoos are currently the best and the most expensive breeding institutions, their focus on endangered species could be highly beneficial for an ex situ conservation. Regional Parrot TAGs (Taxon Advisory Groups) already support these priorities in their suggestions for the establishment of parrot studbooks [51].

The finding that perceived beauty of a parrot species enhances its likelihood to be kept in zoos may have serious consequences for conservation biology. It further corroborates the hypothesis that the fate of the species may be considerably affected by its core attractiveness to humans. Thus, contemporary conservation biology would benefit from focusing on animal beauty and human evolutionary psychology. Moreover, it is a demonstration that the animal morphological traits affecting human behavior towards these animals may affect success of not only individuals, but also species (when facing species selection caused by human pressure).

## References

[1] del Hoyo J, Elliott A, Sargatal J (1997). Handbook of the birds of the world. Vol. 4.. Sandgrouse to cuckoos.

[2] Pepperberg IM (1994). Evidence for numerical competence in an African grey parrot (*Psittacus erithacus*).. J Comp Psychol.

[3] Funk M (2002). Problem solving skills in young yellow-crowned parakeets (*Cyanoramphus auriceps*).. Anim Cogn.

[4] Pepperberg IM (1999). The Alex studies: cognitive and communicative abilities of grey parrots..

[5] Huber L, Gajdon GK (2006). Technical intelligence in animals: the kea model.. Anim Cogn.

[6] Emery NJ, Seed AM, von Bayern AM, Clayton NS (2007). Cognitive adaptations of social bonding in birds.. Philos Trans R Soc Lond B Biol Sci.

[7] Pepperberg I (2006). Grey parrot numerical competence: a review.. Anim Cogn.

[8] Borsari A, Ottoni E (2005). Preliminary observations of tool use in captive hyacinth macaws (*Anodorhynchus hyacinthinus*).. Anim Cogn.

[9] Zentall TR (2004). Action imitation in birds.. Learn Behav.

[10] Mui R, Haselgrove M, Pearce J, Heyes C (2008). Automatic imitation in budgerigars.. Proc R Soc Lond B Biol Sci.

[11] Anderson P (2003). A bird in the house: an anthropological perspective on companion parrots.. Soc Anim.

[12] IUCN Red List of Threatened Species (accessed 2009). World Conservation Union, http://www.iucnredlist.org.

[13] Collar NJ, Juniper AT, Beissinger SR, Snyder NFR (1992). New world parrots in crisis: solutions from conservation biology..

[14] Beissinger SR, Bucher EH (1992). Can parrots be conserved through sustainable harvesting?. BioScience.

[15] Wright TF, Toft CA, Enkerlin-Hoeflich E, Gonzalez-Elizondo J, Albornoz M (2001). Nest poaching in neotropical parrots.. Conserv Biol.

[16] González JA (2003). Harvesting, local trade, and conservation of parrots in the Northeastern Peruvian Amazon.. Biol Conserv.

[17] Pain DJ, Martins TLF, Boussekey M, Diaz SH, Downs CT (2006). Impact of protection on nest take and nesting success of parrots in Africa, Asia and Australasia.. Anim Conserv.

[18] Herrera M, Hennessey B (2007). Quantifying the illegal parrot trade in Santa Cruz de la Sierra, Bolivia, with emphasis on threatened species.. Bird Conserv Int.

[19] Sanz V, Grajal A (1998). Successful reintroduction of captive-raised yellow-shouldered Amazon parrots on Margarita Island, Venezuela.. Conserv Biol.

[20] Collazo JA, White TH, Vilella FJ, Guerrero SA (2003). Survival of captive-reared hispaniolan parrots released in Parque Nacional del Este, Dominican Republic.. Condor.

[21] Brightsmith D, Hilburn J, del Campo A, Boyd J, Frisius M (2005). The use of hand-raised psittacines for reintroduction: a case study of scarlet macaws (*Ara macao*) in Peru and Costa Rica.. Biol Conserv.

[22] Snyder NFR, Koenig SE, Koschmann J, Snyder HA, Johnson TB (1994). Thick-billed parrot releases in Arizona.. Condor.

[23] Munn CA, Luescher A (2006). Parrot conservation, trade, and reintroduction.. Manual of parrot behavior.

[24] Jeggo DF, French H, Bellingham L, Copsey J, Fidgett AL (2000). Breeding programme for St Lucia amazon.. Int Zoo Yearb.

[25] Brock MK, White BN (1992). Application of DNA fingerprinting to the recovery program of the endangered Puerto Rican parrot.. P Natl Acad Sci U S A.

[26] Meyers JM (1996). Evaluation of 3 radio transmitter and collar designs for Amazona.. Wildl Soc Bull.

[27] White TH, Collazo JA, Viella JF (2005). Survival of captive-reared Puerto Rican parrots released in the Caribbean national forest.. Condor.

[28] Sheppard C (1995). Propagation of endangered birds in US institutions: how much space is there?. Zoo Biol.

[29] Balmford A, Mace GM, Leader-Williams N (1996). Designing the ark: setting priorities for captive breeding.. Conserv Biol.

[30] Ward PI, Mosberger N, Kistler C, Fischer O (1998). The relationship between popularity and body size in zoo animals.. Conserv Biol.

[31] Balmford A (2000). Separating fact from artifact in analyses of zoo visitor preferences.. Conserv Biol.

[32] Ward PI (2000). Zoo visitor preferences: reply to balmford.. Conserv Biol.

[33] Haahr M (accessed 2007) True Random Numbers Generator - Integers/Sequences. http://www.random.org/

[34] BirdLife International (accessed 2008) The BirdLife checklist of the birds of the world, with conservation status and taxonomic sources. Version 1. http://www.birdlife.org/datazone/species/downloads/BirdLife_Checklist_Version_1.zip

[35] Forshaw JM, Knight F (2006). Parrots of the world: an identification guide..

[36] Juniper T, Parr M (2003). Parrots: a guide to the parrots of the world..

[37] Marešová J, Frynta D (2008). Noah's Ark is full of common species attractive to humans: the case of boid snakes in Zoos.. Ecol Econ.

[38] Marešová J, Landová E, Frynta D (2009). What makes some species of milk snakes more attractive to humans than others?. Theory Biosci.

[39] Schmitz C et al. (accessed November 2007) LimeSurvey - The open source survey application. Version 1.53plus. http://www.limesurvey.org

[40] International Species Information System (ISIS) database (accessed 2008) http://www.isis.org

[41] Flesness NR, Lukens DR, Porter SB, Wilson CR, Grahn LV (1995). ISIS and studbooks, very high census correlation for the North American zoo population: a reply to Earnhardt, Thompson, and Willis.. Zoo Biol.

[42] Arndt T (2004). Lexicon of Parrots 2.0 (CD-ROM Version)..

[43] Robiller F (1992). Handbuch der Vogelpflege.. Über die Papageien der Welt in drei Bänden.

[44] Wright TF, Schirtzinger EE, Matsumoto T, Eberhard JR, Graves GR (2008). A multilocus molecular phylogeny of the parrots (Psittaciformes): support for a Gondwanan Origin during the Cretaceous.. Mol Biol Evol.

[45] StatSoft (accessed 2001) Statistica, Version 6.0. http://www.statsoft.com

[46] SPSS Inc (accessed 2007) Spss, version 16.0. http://www.winwrap.com

[47] Marešová J, Krása A, Frynta D (2009). We all appreciate the same animals: cross-cultural comparison of human aesthetic preferences for snake species in Papua New Guinea and Europe.. Ethology.

[48] Frynta D, Marešová J, Landová E, Lišková S, Šimková O, Columbus AM, Kuznetsov L (2009). Are animals in zoos rather conspicuous than endangered?. Endangered species: new research.

[49] Soulé M, Gilpin M, Conway W, Foose T (1986). The millenium ark: how long a voyage, how many staterooms, how many passengers?. Zoo Biol.

[50] WAZA (2005). Building a future for wildlife - the world zoo and aquarium conservation strategy..

[51] Wilkinson R (2000). An overview of captive-management programmes and regional collection planning for parrots.. Int Zoo Yearb.

